# α-Synuclein arginylation in the human brain

**DOI:** 10.1186/s40035-022-00295-0

**Published:** 2022-04-08

**Authors:** Jun Zhao, Buyan Pan, Marie Fina, Yun Huang, Marie Shimogawa, Kelvin C. Luk, Elizabeth Rhoades, E. James Petersson, Dawei W. Dong, Anna Kashina

**Affiliations:** 1 Department of Biomedical Sciences, University of Pennsylvania School of Veterinary Medicine, Philadelphia, Pennsylvania 19104 USA; 2 Department of Chemistry, University of Pennsylvania School of Arts and Sciences, Philadelphia, Pennsylvania 19104 USA; 3 Center for Neurodegenerative Disease Research, Department of Pathology and Laboratory Medicine, University of Pennsylvania Perelman School of Medicine, Philadelphia, Pennsylvania 19104 USA

**Keywords:** Arginylation, Neurodegeneration, Aging, α-Synuclein

## Abstract

**Background:**

Alpha-synuclein (α-syn) exhibits pathological misfolding in many human neurodegenerative disorders. We previously showed that α-syn is arginylated in the mouse brain and that lack of arginylation leads to neurodegeneration in mice.

**Methods:**

Here, we tested α-syn arginylation in human brain pathology using newly derived antibodies in combination with Western blotting, biochemical assays, and experiments in live neurons.

**Results:**

We found that α-syn was arginylated in the human brain on E46 and E83, two sites previously implicated in α-syn pathology and familial cases of Parkinson’s disease. The levels of arginylation in different brain samples ranged between ~ 3% and ~ 50% of the total α-syn pool, and this arginylation nearly exclusively concentrated in the subcellular α-syn fraction that sedimented at low centrifugation speeds and appeared to be simultaneously targeted by multiple posttranslational modifications. Arginylated α-syn was less susceptible to S129 phosphorylation and pathological aggregation in neurons. The arginylation level inversely correlated with the overall α-syn levels and with patient age, suggesting a possible causal relationship between arginylation decline and α-syn-dependent neuropathology.

**Conclusion:**

We propose that α-syn arginylation constitutes a potential neuroprotective mechanism that prevents its abnormal accumulation during neurodegeneration and aging in the human brain.

**Supplementary Information:**

The online version contains supplementary material available at 10.1186/s40035-022-00295-0.

## Background

Alpha-synuclein (α-syn) exhibits pathological misfolding and aggregation in human neurodegenerative disorders that are collectively called synucleinopathies [1]. One of the most prevalent diseases in this group is Parkinson’s disease (PD), which is characterized by the formation of Lewy neurites and Lewy bodies (LBs) – large α-syn aggregates that contribute to neuronal loss, dementia, and death [2]. It is well established that LB formation and maturation are associated with multiple posttranslational modifications (PTMs) [3, 4], including, most prominently, phosphorylation of α-syn at S129, which is generally believed to be involved in PD pathology [5]. However, the exact role of this and other PTMs in regulating α-syn misfolding and aggregation is not well understood.

Protein arginylation mediated by arginyltransferase ATE1 is a PTM of emerging biological significance that consists of transfer ribonucleic acid (tRNA)-dependent transfer of Arg (R) to proteins and has been implicated in many key physiological events in vivo [6, 7]. ATE1 can modify proteins either N-terminally or on the side chains of the acidic residues, i.e., Asp (D) or Glu (E) [8]. Previous work from our lab uncovered that α-syn is a highly efficient target for ATE1 in vitro, and that in mouse brain α-syn is arginylated at E46 and E83 [9], two sites that have been previously implicated in α-syn function and PD pathology in human patients [10, 11]. Mice lacking ATE1 in the brain develop symptoms of neurodegeneration, suggesting that arginylation plays a role in normal brain function and may act specifically via α-syn [9].

Here, we tested whether E46 and E83 arginylation targets α-syn in the human brain, and whether this arginylation correlates with any physiological changes in the patients, such as disease status or age. We also tested the potential interplay between α-syn arginylation and S129 phosphorylation, another PTM previously implicated in PD pathology. Our results uncover a potential new mechanism of α-syn regulation in the human brain and propose functional implications of arginylation in neurodegeneration and aging.

## Materials and methods

### Materials

Frozen postmortem brain samples of human frontal cortex were from patient brain donors who underwent autopsy at the Center for Neurodegenerative Disease Research (CNDR) at the University of Pennsylvania between 1992 and 2016 [12]. Detailed clinical characteristics (age, sex, diagnosis) are listed in Additional file 1: Table S1. All antibodies used in this project are listed in Additional file 1: Table S2.

### Synthesis of fluorenylmethyloxycarbonyl (Fmoc)-Glu(Arg(Pbf)-OtBu)-OH

To generate branched peptrides mimicking arginylated sites on α-syn, we synthesized Fmoc-Glu(Arg(Pbf)-OtBu)-OH, an arginylated Glu derivative to be used to synthesize the Glu-arginylated peptides. To do this we first generated a precursor, Fmoc-Glu(Arg(Pbf)-OtBu)-OAll. The procedure for precursor generation and final synthesis was as follows. Two hundred and five milligrams of Fmoc-Glu-OAll (0.5 mmol) were dissolved in 15 ml tetrahydrofuran (THF) in a flask and the flask was cooled to -20 °C. Next, 60 μl of N-methylmorpholine (0.5 mmol) was added to this flask, followed by dropwise addition of 65 μl isobutyl chloroformate (0.5 mmol). The mixture was stirred for 10 min, and then 259 mg Arg(Pbf)-OtBu·HCl (0.5 mmol) and additional 60 μl of N-methylmorpholine were added. The resulting slurry was stirred at − 20 °C for 1 h and then at room temperature for 3 h. The precipitate was filtered off and the filtrate was evaporated under reduced pressure. The residue was dissolved in 30 ml EtOAc and then washed with NaHCO_3_ and brine. The organic layer was dried over MgSO_4_ and evaporated to give rise to 460 mg oil, which was used directly for the next step. The obtained Fmoc-Glu(Arg(Pbf/OtBu)-OAll (0.5 mmol) was dissolved in 5 ml dichloromethane (DCM). To this flask was added 15 mg Pd(PPh3)4 (0.0125 mmol, 2.5% mol) and 617 μl PhSiH3 (5 mmol) under argon. The mixture was stirred for 4 h at room temperature and then evaporated under reduced pressure. The residue was re-dissolved in 20 ml DCM and washed with NaHCO_3_ and brine. The organic layer was dried over MgSO_4_ and evaporated to dryness. Further purification using silica gel gave rise to 375 mg pure Fmoc-Glu(Arg(Pbf)-OtBu)-OH with a total yield of 45%.

The compounds were verified by matrix assisted laser desorption/ionization (MALDI) to determine their mass, as follows: Fmoc-Glu(Arg(Pbf)-OtBu)-OAll: [MH]^+^ 874.29; Fmoc-Glu(Arg(Pbf)-OtBu)-OH: [MH]^+^ 834.3750.

### Synthesis of E46- and E83-arginylated α-syn peptides used as antigen for antibody generation

The arginylated peptides CVGSKTKE^R^GVVH and CAVAQKTVE^R^GAG were synthesized manually using standard Fmoc-based strategy with modification as reported in [13]. Briefly, 20 mg 2-chlorotrityl resin (100–200 mesh, 1.5 mmol substitution/g) was swelled in dry DCM for 1 h. Then the DCM was removed and the first Fmoc-amino acid (1 equiv.) in 2 ml DCM and DIPEA (4 equiv.) were added to the resin. Upon mixing for 40 min, the resin was washed with DMF for four times. The resin was then capped by washing 3 times with DCM/MeOH/DIPEA (volume ratio 17:2:1), 3 times with DCM, and 3 times with DMF. The Fmoc group was removed by adding 2 ml of 20% peperidine/DMF and stirring for 20 min. The amount of coupled amino acid was evaluated by the Fmoc content in the deprotection solution using absorbance at 300 nm (extinction coefficient: 7800 cm^−1^ M^−1^). Subsequent amino acids were coupled by adding Fmoc-amino acid (5 equiv.) that was activated by HBTU (5 equiv.) and DIPEA (10 equiv.) and stirring for 30 min. For the on-resin coupling of Fmoc-Glu(Arg(Pbf)-OtBu)-OH, 2 equiv. of Fmoc-Glu(Arg(Pbf)-OtBu)-OH in DMF was activated by 2 equiv. of HBTU and 4 equiv. of DIPEA and then mixed with resin for 2 h. After further elongation, the peptides were cleaved from resin by treatment with 90%TFA/5%TIPS/5%DCM for 1.5 h. The cleavage solution was pooled into cold ether and the precipitate was collected and purified by reverse-phase HPLC using VyDAC C18 column and 0.1% TFA/0.1% acetonitrile as the mobile phase. The purity was checked by analytic HPLC (Additional file 2: Dataset 1) and identity was confirmed by mass spectrometry (Additional file 2: Dataset 1).

MALDI:

- CVGSKTKE^R^GVVH: Calcd. [MH]^+^ 1399.75, found. 1399.69;
- CAVAQKTVE^R^GAG: Calcd. [MH]^+^ 1189.66, found. 1289.93.

### Generation of synthetic arginylated α-syn variants

Synthetic arginylated full-length α-syn variants were generated as described in [13].

### Immunoblotting

Frozen tissues were grounded in liquid nitrogen using a mortar and pestle until fine powder was formed. The pulverized tissue powder was then weighed and lysed directly in 4 × SDS loading buffer (1:10 *w*/*v* ratio), followed by boiling for 10 min. Then 2.5 μl of each sample was loaded on 15% SDS–polyacrylamide gels (PAGE) and transferred to a 0.2-μm nitrocellulose membrane at 250 mA for 20 min. Blots were blocked in 3% BSA in TBST, then incubated with primary antibodies (Additional file 1: Table S2) at 4 °C overnight. Then the membranes were incubated with secondary antibodies (1:5000 dilution) conjugated to IRDye800 or IRDye700 and images were acquired using the Odyssey Imaging System. For comparison of arginylated α-syn levels across different human brain samples, GAPDH was used as an internal loading control.

### Fractionation of soluble and insoluble α-syn

The tissue powder was lysed in reaction buffer (containing 100 mM HEPES, pH 7.2, 50 mM KCl, 1 mM MgCl_2_, 1 mM EGTA, and 1 mM DTT, with Sigma protease inhibitor cocktail added before use) at 1:10 *w*/*v* ratio, and then centrifuged at 13,000 r/min in a tabletop microfuge at 4 °C for 30 min. The supernatant and pellet were collected as soluble and insoluble pool for Western blotting analysis. The supernatant was mixed at 1:1 ratio with 4× SDS loading buffer. The pellet was resuspended in 4× SDS loading buffer equal to the volume of the original extract, and both samples were boiled for 10 min prior to loading on the gel. Then 6 µl of pellet and 20 µl of supernatant were loaded on 10% bis–tris gels for mass spectrometry or 15% SDS-PAGE for Western blot.

### In vitro phosphorylation of α-syn using Polo-like kinase 2 (PLK2)

For each reaction, PLK2 solution (Catalog Number: PV4204, ThermoFisher Scientific, Waltham, MA) was mixed with recombinant α-syn at a ratio of 2 µl PLK2 per 72 µg α-syn in the reaction buffer containing 25 mM HEPES, pH 7.2, 50 mM NaCl, 20 mM MgCl_2_, 2 mM DTT, and 1 mM ATP freshly added from a frozen stock. For the reaction, components were mixed on ice in the following order: final reaction buffer, α-syn, followed by PLK2, and the mixture was then transferred to 30 °C for 2 h. For negative controls, ATP or PLK2 was omitted from the reaction, as indicated elsewhere. After completion of the reaction, α-syn phosphorylation was detected by Western blots using the S129 antibody.

### α-Syn aggregation assays in cultured primary neurons

Primary neurons were isolated from C57-Bl6 mouse neonatal brain as described [14], and incubated with non-arginylated α-syn or its arginylated variants (E46, E83, and double) as described [15]. Detection of α-syn inclusions was performed using the S129 antibody, and the inclusion fluorescence and shape were quantified using the “integrated morphometry analysis” function in the Metamorph imaging software (Molecular Devices, Downington, PA). The original images were uniformly processed using the “background subtraction” function in the Metamorph imaging software.

### Negative staining electron microscopy

Non-arginylated or 5%-arginylated α-syn fibrils (E46, E83, and double) were deposited on formvar-coated grids, stained with 2% *w*/*v* uranyl acetate in water, and visualized by transmission electron microscopy.

### Image analysis and quantification

Western blots were quantified by direct measurements of the secondary antibody fluorescence using Odyssey gel imager (LiCor, Lincoln, NE). Quantification of the band intensity over the background was calculated using the Odyssey gel imaging software integrated with the instrument.

For description of intracellular S129-positive aggregates, the following parameters were measured:

1. “total intensity” (following the command name in the Metamorph imaging software) measures the total gray level of each aggregate, thresholded against the background; this parameter directly depends on the area and density of the aggregate and thus quantitatively reflects the aggregate size.
2. “average intensity” (following the command name in the Metamorph imaging software) measures the gray levels (equaling fluorescence intensity) per unit area, and this reflects the density of the aggregate, as more densely packed aggregate would have higher fluorescence per unit area.

### Statistical analysis

**Fig. 1:**
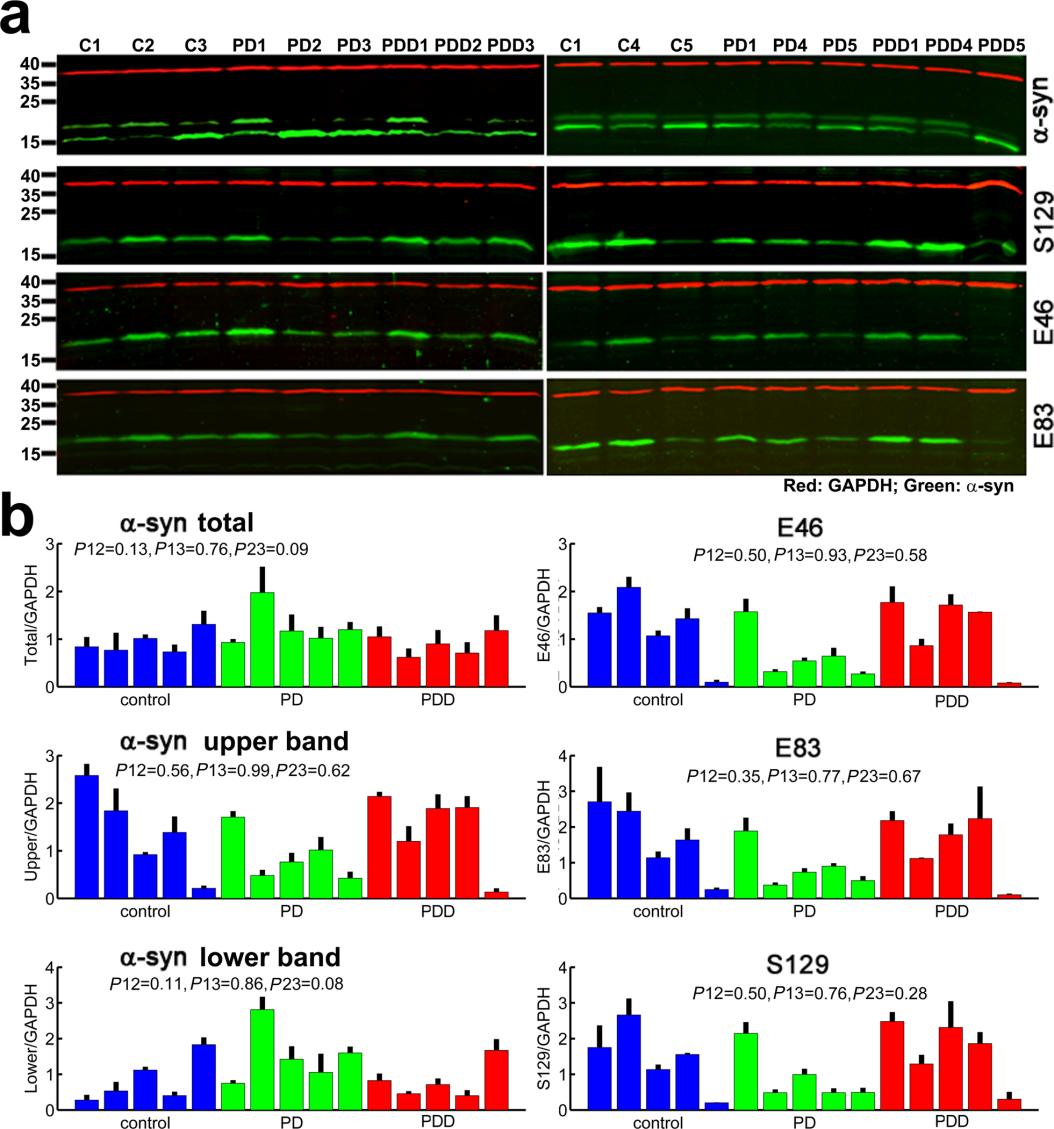
E46- and E83-arginylated α-syn are present in the human frontal cortex and show varied levels in PD patients and healthy controls. **a** Representative immunoblots of total α-syn, as well as α-syn modified by phosphorylation at S129 or arginylation at E46 or E83. **b** Quantification of α-syn signal from all antibodies in all samples; for total α-syn, signals from both bands of the doublet in the ~ 14 kDa range were added, and the upper and lower bands of the doublet were also quantified separately. Error bars represent SEM from triplicate runs of the same samples. C, control; PD, Parkinson’s disease; PDD, Parkinson disease with dementia. *P*-value comparisons between control and PD (*P*12), control and PDD (*P*13), as well as PD and PDD (*P*23) are shown on top of each panel, calculated by two-tailed Welsh *t*-test. See Table S1 for further details on the patient samples

Calculations for Figs. 1, 3, S3, S4, and S5 were performed using MATLAB software, and for the other figures using the Graphpad Prism software package (version 8.0). For the Graphpad Prism analysis, the data are presented as mean ± standard deviation (SD), unless otherwise indicated, and statistical comparisons between different groups were first performed using one-way ANOVA analysis, followed by two-tailed student’s *t*-test between two groups with the *P*-value corrected by Bonferroni test.

## Results

### α-Syn is arginylated at E46 and E83 in the human brain

Our previous work showed that α-syn is arginylated in the mouse brain on two conserved sites, E46 and E83, which are targeted for arginylation within the intact non-proteolyzed α-syn protein [9]. Both of these sites are conserved between mouse and human α-syn, and both have previously been implicated in α-syn pathology, including the familial E46K mutation in PD patients [3, 11]. To test whether E46 and E83 arginylation occur in human synucleinopathy and to dissect their potential roles in pathogenesis, we raised rabbit polyclonal antibodies against synthetic peptides corresponding to the α-syn side-chain arginylated at E46 and E83 (Additional file 1: Fig. S1a). These antibodies were specific for the corresponding arginylated peptides and full-length synthetic arginylated α-syn, and showed no cross-reactivity with each other, or with non-arginylated α-syn protein and peptides (Additional file 1: Fig. S1a, b).

We next used these antibodies to probe extracts from the frontal cortex samples of patients, obtained from the biobank at the Center for Neurodegenerative Disease Research (CNDR) at the University of Pennsylvania. We analyzed healthy controls, as well as patients with PD and PD-related dementia (PDD, characterized by severe neuronal loss and pathological α-syn aggregation). Strikingly, all brain samples showed prominent reactivity with both E46 and E83 antibodies (Fig. 1), suggesting that the human α-syn is arginylated in vivo at both E46 and E83.

Several interesting trends were observed by comparisons of the patient samples (Fig. 1a, b). First, the levels of both E46 and E83 arginylation strongly varied between samples, suggesting that arginylation in different individuals can target vastly different fractions of the intracellular α-syn pool. Second, the levels of arginylation of E46 and E83 showed a similar trend, with a comparatively higher or a lower antibody signal in the same samples, suggesting that α-syn arginylation in these samples is high or low as a whole, without apparent selectivity between the two arginylation sites. Notably, the same trends were also observed with antibodies against α-syn phosphorylated at S129, suggesting that all three modifications co-elevate in some samples compared to others and may potentially interact with each other.

### E46 and E83 arginylation targets the α-syn pool that sediments at lower centrifugation speeds and is also enriched in S129 phosphorylation

**Fig. 2:**
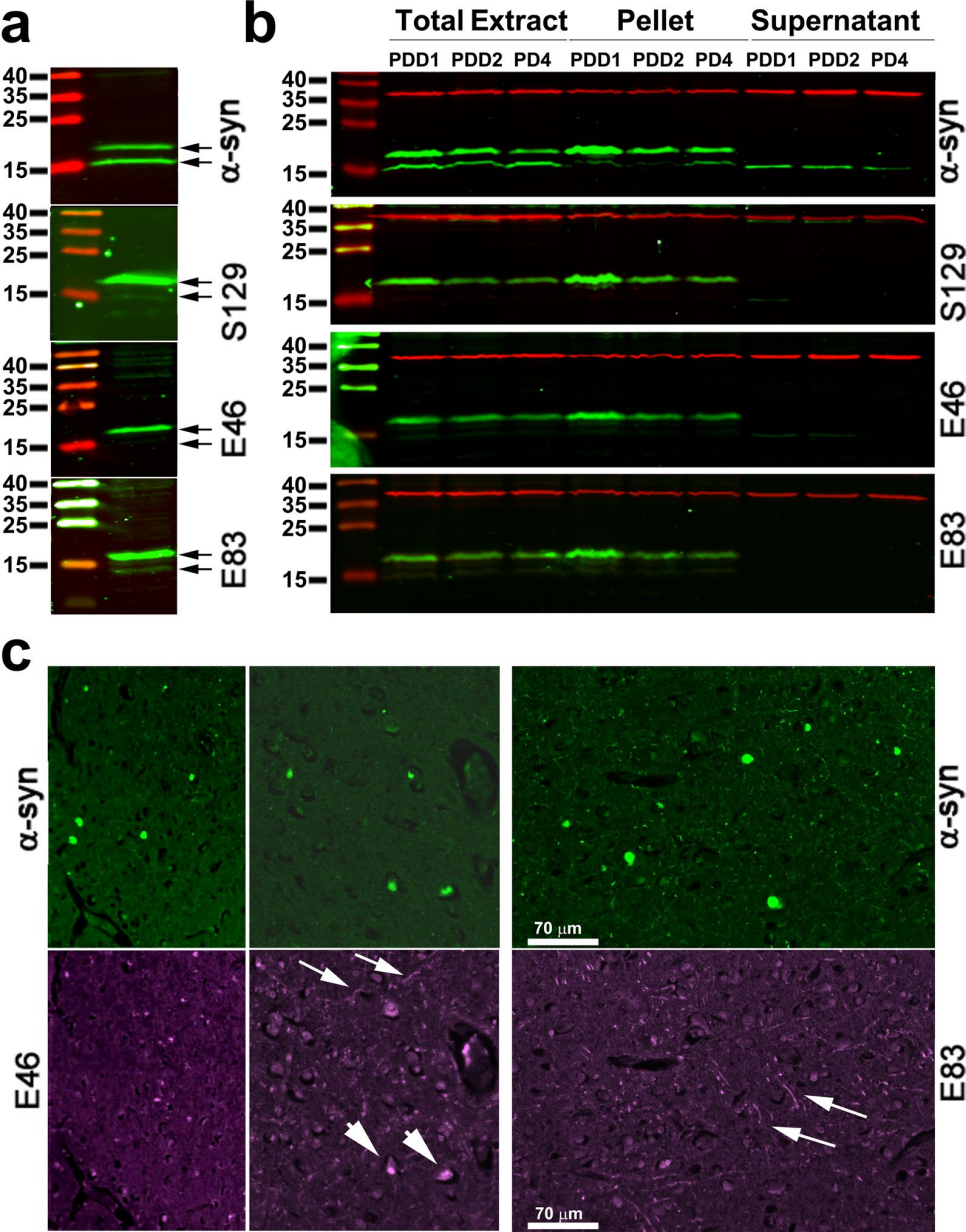
E46 and E83 arginylation targets the α-syn pool that sediments at lower centrifugation speeds in extracts from the frontal cortex, but does not strongly colocalize with Lewy bodies in the frontal cortex sections from human PD patients. **a** Magnification of a representative Western blot of the sample C1 probed with the total and modified α-syn antibodies, showing the doublet α-syn bands recognized by the total α-syn antibody and the relative position of the single band preferentially recognized by the antibodies to the modified α-syn variants as the upper band in this doublet. **b** Representative Western blots from the total brain extract, pellet, and supernatant fractions. Bands of heavily modified α-syn were preferentially found in the pellet, indicating its incorporation into the insoluble α-syn pool. **c** Representative images of human brain sections showing prominent LB formation, stained with antibodies to total α-syn, as well as the antibodies against E46- and E83-arginylated variants. LBs were prominently seen as large green dots in the top set of images stained with total α-syn antibodies. Most of these LBs show no specific staining with E83, and only occasional aggregates were visualized with E46 antibodies (short arrows). Both E46 and E83 antibodies also stained fibrillar aggregates (long arrows)

During the experiments described above, we noticed that the single band on the SDS-PAGE recognized by the antibodies for arginylated and S129-phosphorylated α-syn ran somewhat higher on the gel than the expected 14 kDa. At the same time, the total α-syn antibodies recognized a doublet in the ~ 14 kDa range, with the upper band in the doublet matching the position of the band positive for both arginylated and phosphorylated α-syn (Fig. 2a). Since SDS gel shifts often happen after proteins have undergoen PTMs, including phosphorylation [16], we hypothesized that the upper band represents a subset of α-syn that is simultaneously targeted by multiple PTMs.

First, to test if this upper band indeed represents α-syn, we excised bands of matching molecular weight from an SDS gel and identified protein composition in these bands by mass spectrometry to confirm the presence of α-syn as a major protein in both bands (Additional file 1: Fig. S2). Next, to test whether modified and unmodified α-syn are enriched in the same subcellular fractions, we fractionated human brain extracts by centrifugation at 13,000 g and tested the supernatant and pellet from this step by Western blot. Strikingly, the majority of the upper band, detectable by pS129, E46-Arg, and E83-Arg antibodies, was found in the pellet, while the lower band remained in the supernatants (Fig. 2B). Thus, all three PTMs nearly exclusively target the α-syn pool that sediments at lower speeds, a subcellular fraction that typically contains intracellular organelles and larger protein aggregates.

During PD, pathologically misfolded insoluble α-syn often incorporates into LBs, large α-syn-rich protein aggregates that are known to be targeted by multiple PTMs as a hallmark feature of PD pathology [2]. Previous studies have associated LBs with pS129 staining [17], even though the exact role of S129 phosphorylation of α-syn in PD pathology remains controversial [8, 18]. To test whether E46 and E83 arginylation targets LBs, we co-stained brain sections from human PD patients with antibodies to total α-syn and either E46-Arg or E83-Arg α-syn (Fig. 2c). Total α-syn staining revealed the presence of large aggregates clearly visible in different areas of the sections. However, these aggregates did not show any enrichment in E83-Arg staining. In the case of E46-Arg, most of the aggregates were also not stained, but some of the larger aggregates, with morphology characteristic for the later stages of LB formation, were somewhat highlighted with E46-Arg, suggesting that E46-arginylated α-syn penetrates the LBs over time, possibly during LB maturation. Notably, both E46-Arg and E83-Arg antibodies also showed some fibrillar staining patterns, suggesting that these modifications target α-syn-containing structures in the brain other than LBs. It is unclear if this LB-independent α-syn pool is involved in normal brain function and/or plays a neuroprotective, rather than pathological, role.

### α-Syn E46 and E83 arginylation in the human brain inversely correlates with the total α-syn levels and prominently decreases with age

**Fig. 3:**
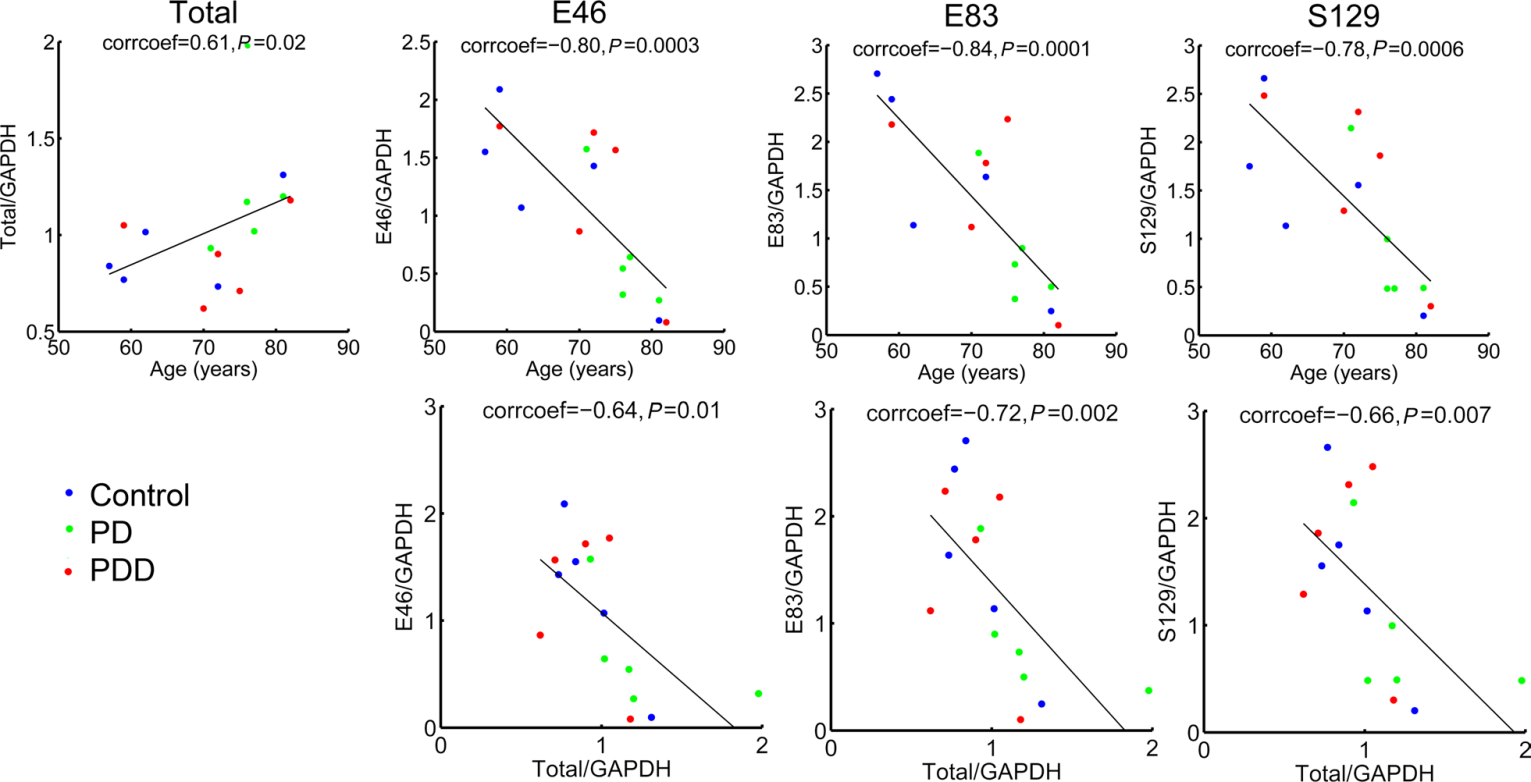
E46 and E83 arginylation in the human frontal cortex inversely correlates with α-syn levels and decreases with patient age. Correlation plots between different antibody signals calculated from the same datasets as those shown in Fig. 1. Spearman correlation coefficients and *P *values are shown on top of each plot. See Additional file 1: Fig. S3 and S4 for additional plots

Next, we used the quantification data (Fig. 1) to analyze correlations between the Western blot signals for E46/E83 arginylation, S129 phosphorylation, and total α-syn in different patients. We quantified total α-syn in each sample as the sum of the upper and lower bands in the doublet (Fig. 3), and the upper and lower bands separately, corresponding to the modified and unmodified α-syn fractions, respectively (Additional file 1: Fig. S3).

This analysis revealed several interesting trends. First, while the total α-syn levels increased with the patient age, E46/E83 arginylation as well as S129 phosphorylation showed a prominent decrease in older patients (Fig. 3, top row). This decrease was due almost exclusively to the decrease in the upper band of the doublet, while the lower band followed the total α-syn trends (Additional file 1: Fig. S4). Interestingly, within the samples we tested, no significant correlation was seen between the levels of each PTM and the PD or PDD diagnosis (with *P* values for comparison between the groups ranging at 0.5 and above, Fig. 1b), suggesting that brain aging, rather than PD diagnosis, is the primary factor in α-syn arginylation.

Both arginylation and S129 phosphorylation inversely correlated with the total α-syn levels in different patients (Fig. 3, bottom row), suggesting that all three modifications are antagonistic to α-syn accumulation in the brain, a proposed major factor in α-syn-driven neurodegeneration [19, 20]. All three modifications showed a strong positive correlation with the upper band in the doublet and a strong inverse correlation with the lower band (Additional file 1: Fig. S3). Consistent with this, all three modifications showed similar correlation trends, strongly suggesting that they were positively correlated with each other (Additional file 1: Fig. S3). All these changes may potentially reflect functional interactions between arginylation, phosphorylation, and changes of α-syn states in the brain. Of note, none of these changes correlated with a total change in ATE1 level in these samples (Additional file 1: Fig. S5), suggesting that the changes in arginylation of α-syn at E46 and E83 in different brain specimens are not a direct consequence of the altered availability of arginyltransferase.

These data collectively demonstrate that α-syn E46 and E83 arginylation targets a specific α-syn pool in the human brain in a manner antagonistic to overall α-syn accumulation and correlates with changes in the brain’s physiological state during aging.

### Quantification of α-syn arginylation in the human brain

**Fig. 4:**
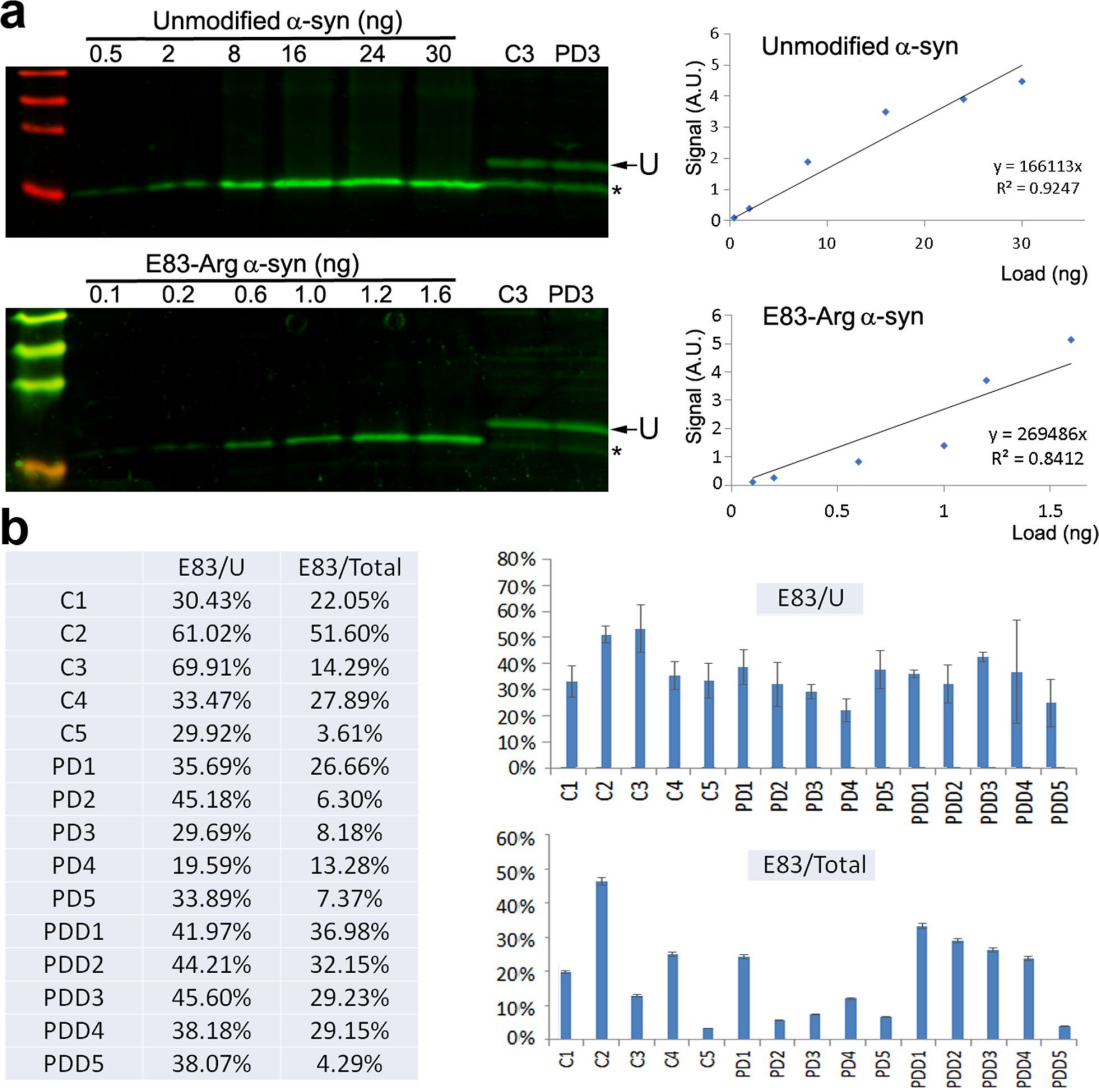
Quantification of E83 arginylation as a fraction of the total α-syn levels in the human frontal cortex. **a** Representative Western blot images (left) and calibration curves (right), of standard unmodified and E83-arginylated α-syn. **b** Quantification of the fraction of E83-arginylated α-syn as the percentage of the upper band (U, marked with arrows in both gels) and of the total α-syn in the preparation (the sum of upper and lower bands marked with arrow and asterisk, respectively), calculated by running the calibrations. Percentage of arginylated α-syn to the upper band (U) and the total are shown both as a table (left) and as a chart (right)

To quantify the percentage of arginylated α-syn in the human brain in individual samples, we used synthetic E83-Arg α-syn as a protein standard to calibrate the signal, alongside the unmodified α-syn. We first ran calibration curves to directly quantify the amount of modified and unmodified α-syn in the standard lanes as the function of the Western blot signal and determine the linear range (Fig. 4a). Next, we ran some of these linear range standards at known loads alongside the human patient brain samples to directly measure the actual amount of total and E83-Arg α-syn in each brain sample. These measurements enabled us to calculate the percentage of E83-arginylated α-syn in each sample.

While these percentages varied across different samples, they were remarkably high overall (Fig. 4b), ranging from several percent in some samples to up to ~ 70% of the upper gel band containing the highly modified α-syn pool (calculated from quantifying the signal in the upper band only and comparing it to the protein standard) and up to ~ 50% of total α-syn. This is, by far, the highest fraction of arginylation ever observed for any protein in vivo.

### E46 and E83 arginylation prevents S129 phosphorylation

Our data so far strongly suggest that arginylation at E46 and E83 targets the same pool of α-syn in the brain as S129 phosphorylation, with the possible exception of the LBs. While the role of S129 phosphorylation on α-syn is still debated in the literature, some lines of evidence suggest that this modification is associated with PD pathology [17]. To determine whether arginylation acts synergistically with S129 phosphorylation, or whether these two modifications are antagonistic, we used synthetic arginylated α-syn standards for in vitro phosphorylation assays with Polo-like kinase 2 (PLK2), which mediates α-syn S129 phosphorylation in vivo [18, 21].

**Fig. 5:**
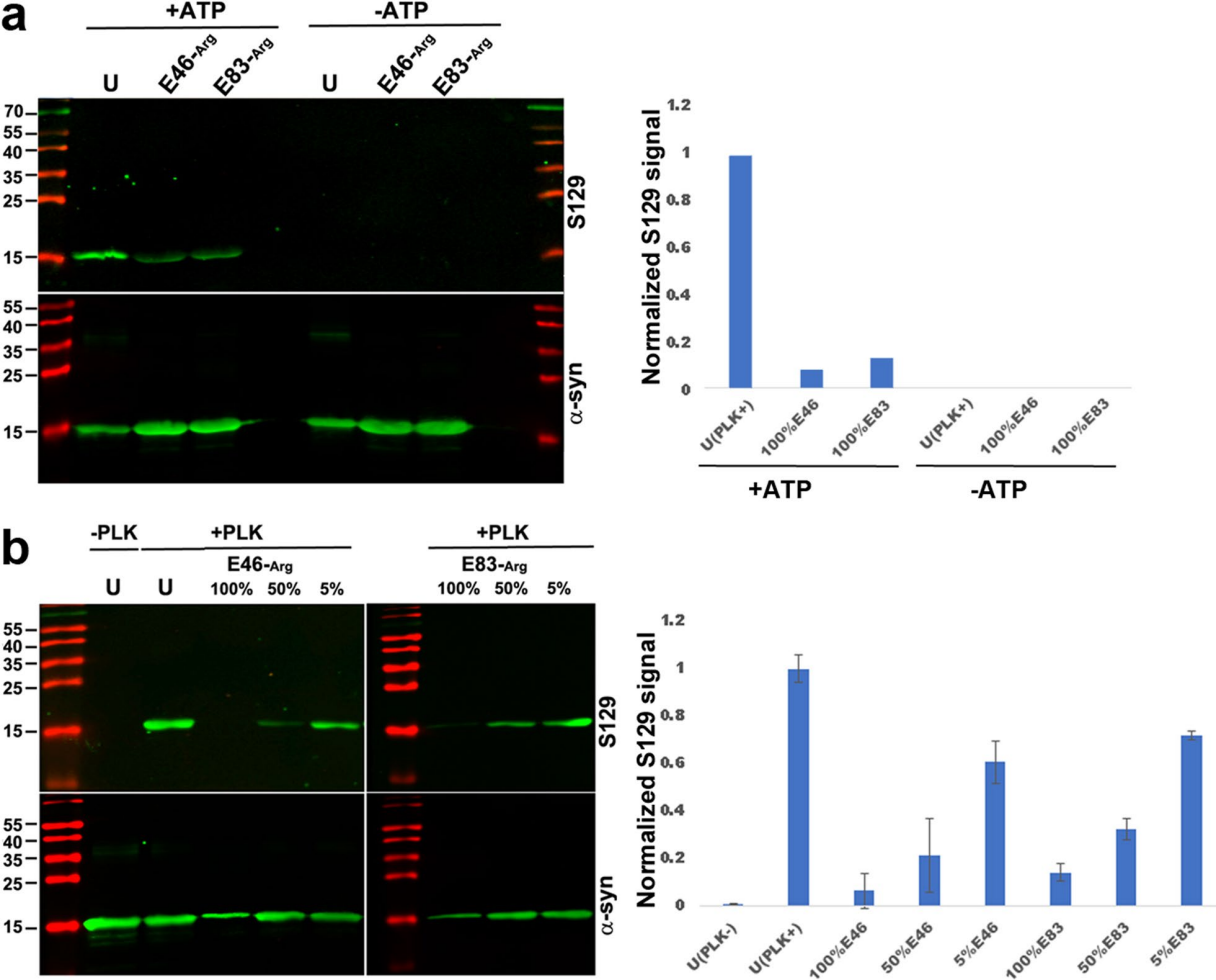
E46 and E83 arginylation prevents S129 phosphorylation. Representative Western blot images (left) and quantification (right) of phosphorylation of unmodified (U) and arginylated α-syn protein standards after the phosphorylation reaction with the addition of components shown on top. **a** α-Syn variants, either unmodified (U) or 100% arginylated at E46 or E83, were incubated with PLK2 kinase in the presence or absence of ATP. Arginylation greatly reduced the efficiency of PLK2 phosphorylation. **b** Phosphorylation of α-syn variants, either unmodified or arginylated at varied percentages as indicated, incubated with or without PLK2 kinase (PLK) as indicated on top. Arginylation interfered with PLK2 phosphorylation in a dose-dependent manner. Band intensities of S129 signal normalized to α-syn load are plotted. Error bars represent SEM, *n* = 3

First, we compared the phosphorylation efficiency of unmodified α-syn with that of α-syn 100% arginylated at E46 or E83. Both types of arginylation greatly reduced the amount of α-syn capable of undergoing phosphorylation (Fig. 5a). Next, we tested the phosphorylation efficiency on partially arginylated α-syn preparations, obtained by mixing unmodified and fully arginylated α-syn at ratios comparable to physiological level (5% and 50%). These assays showed that a higher percentage of arginylation led to a lower phosphorylation efficiency, suggesting that the negative effect of arginylation on phosphorylation was dose-dependent (Fig. 5b). Thus, arginylation and phosphorylation of α-syn have an antagonistic relationship and arginylation at either E46 or E83 could interfere with α-syn phosphorylation at S129.

### Arginylation at E46 and E83 decreases intracellular α-syn aggregation

To test directly whether α-syn arginylation has a neuroprotective role, we tested the ability of arginylated α-syn at E46 or E83, or the E46/E83 double arginylated variant, to seed pathological inclusions in cultured primary neurons freshly isolated from mouse brain. This assay was previously developed to model the formation of LBs and has been shown to be capable of testing the potential pathological properties of α-syn preparations in seeding LB-like intracellular inclusions that are detectable with S129 antibody [15].

**Fig. 6:**
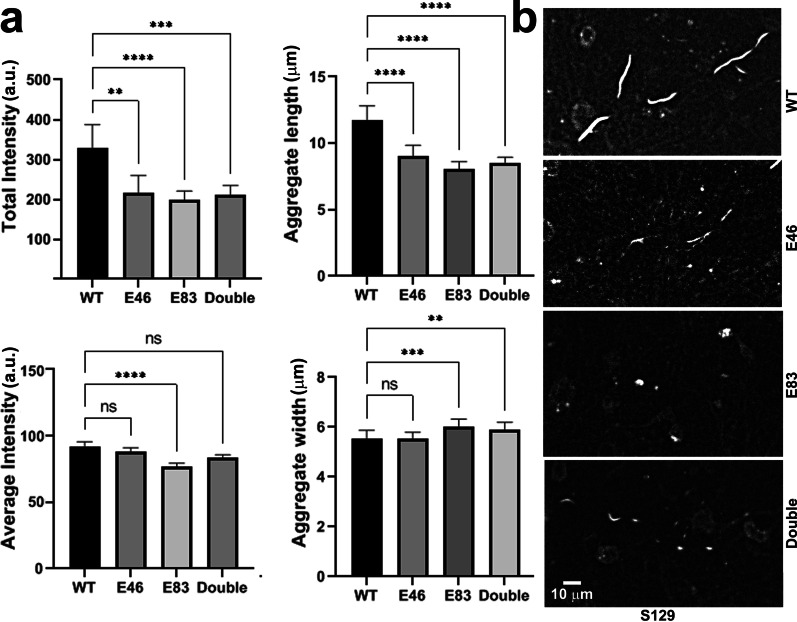
E46 and E83 arginylation decreases intracellular α-syn aggregation. Mouse neurons were incubated with unmodified (WT) α-syn or α-syn arginylated at E46, E83, or both E46/E83 (double), and the formation of LB-like inclusions was examined with S129 antibody. **a** Quantification of the intensity, length, and width of S129-positive inclusions in neuronal cultures, plotted as mean with 95% confidence interval. *n* = 50 independent fields of view. *P* values were calculated by unpaired two-tailed Student's *t*-test, **P < 0.01, ***P < 0.001, ****P < 0.0001. See Additional file 1: Fig. S6 for scatter plots of the data shown in the left panels, plotted as individual data points correlated to relative measurements of the same aggregates. **b** Representative images of inclusions revealed by S129 staining. Scale bar, 10 µm. See Additional file 1: Fig. S7 for broader-field-of-view images of the same aggregates.  Images were processed by uniform background subtraction using Metamorph imaging software

While fibrils prepared from unmodified α-syn formed prominent intracellular pS129-positive inclusions in the soma and neurites, all of the single and double arginylated α-syn variants formed much smaller inclusions, less abundant than those in control (Fig. 6, and Additional file 1: Fig. S6). The total pS129 fluorescence intensity of these inclusions (indicative of their size) was overall ~ 30% lower in arginylated variants compared to unmodified α-syn (Fig. 6a, top left). The average fluorescence intensity (calculated as the total fluorescence intensity divided by the inclusion area, indicative of the density of pS129 monomers within the inclusions) was not changed (Fig. 6a, bottom left). In addition, the inclusions seeded by unmodified α-syn appeared more elongated, while those seeded by arginylated α-syn variants were shorter and more dot-like, a substantial change from the typical morphology observed at the timepoints used (Fig. 6a, right panels). This was confirmed by visual observations (Fig. 6b, and Additional file 1: Fig. S7). We also confirmed that the fibrils pre-added to cells were not substantially different in morphology or aggregation status (Additional file 1: Fig. S8). Thus, arginylation at E46 and E83 interferes with the ability of α-syn to seed LB-like intracellular aggregates and induce neuropathology in cultured neurons.

## Discussion

Our work demonstrated that α-syn was arginylated in the human brain on two conserved, functionally important sites – E46, previously implicated in familial PD, and E83, previously found to be critically important for α-syn pathology [22–24]. We found that E46 and E83 arginylation targeted a different percentage of α-syn in different patients, ranging from a few percent to nearly 50% of the total α-syn pool. Notably, α-syn arginylation on these two sites strongly decreased with age, and it negatively impacted pathological intracellular aggregation of α-syn, suggesting a neuroprotective role of this arginylation.

The familial mutation E46K correlates with PD [22, 23]. Since K is a positively charged amino acid residue that is closest to R in structure and chemical property, one would expect that arginylation on the same site would mimic the effect of this mutation. However, previous studies showed that E46K α-syn is more degradation-resistant and aggregation-prone than wild-type protein [25, 26], and a lack of E46 arginylation also induces α-syn intracellular accumulation [9]. Clearly, E46K mutation is sufficiently different from E46 arginylation, which produce nearly opposite effects on the protein. Given this knowledge, it appears possible that the E46K mutation may exert at least some of its biological effects by preventing arginylation on this site.

While our data suggest that arginylation at E46 and E83 targets the same α-syn pool as phosphorylation at S129, which has been previously proposed to be linked to neuropathology [17], our data also indicate that arginylation prevents or diminishes S129 phosphorylation. It is attractive to suggest that arginylation is an antagonistic mechanism in vivo that counters pathological phosphorylation, and that arginylated α-syn penetrates the misfolding and aggregating α-syn pool with the potential to reverse its pathology and restore its normal function. While direct experiments to test this hypothesis are impossible at present, this idea is supported by our data that arginylation was enriched in the S129-positive insoluble α-syn pool, where it could potentially act as a mechanism to reduce and outcompete the pathological S129 phosphorylation. Also, we found that in the brain, arginylation was primarily present on fibril-like α-syn structures outside LBs, and only E46-arginylated α-syn appeared to enter the especially mature LBs on occasion, possibly to counter their growth and toxicity to neurons. The pathological properties of α-syn fibrils versus other misfolded aggregates (oligomers) are still debated in the literature [27], and some reports suggest that fibrils, as opposed to other aggregates, actually serve a neuroprotective function [28]. We speculate that arginylation may be enriched in such neuroprotective fibrillar aggregates, rather than in the pathology-inducing α-syn pool, and this hypothesis will be elucidated in our future studies.

Interestingly, neither arginylation nor phosphorylation in vitro, separately or together, caused a change in α-syn apparent molecular weight on SDS-PAGE. At the same time, the majority of arginylated and phosphorylated α-syn in the human brain samples appears to cause a gel shift (as seen by comparing the molecular weights of α-syn S129-positive band in Figs. 2 and 5). This strongly suggests that the arginylated/phosphorylated α-syn in the brain is also targeted by additional PTMs, possibly ubiquitination, as well as potentially others, that collectively cause a visible change in α-syn apparent molecular weight. Identifying these PTMs and elucidating their role in α-syn physiology constitute an exciting direction of future research.

While our understanding of protein arginylation as a PTM is still in its early stage, prior studies from our group and others suggest that only a small fraction of each protein is arginylated in vivo at any particular time. For example, less than 1% of β-actin can be arginylated [29], even though this arginylation is likely locally enriched to facilitate its functions in cell migration [30]. In comparison, our current study shows that 3%–50% of total α-syn can be arginylated in different human patients. This constitutes, by far, the highest fraction of arginylation ever observed on any protein in vivo.

Previous studies from our group showed that α-syn enzymatically arginylated in vitro has a reduced ability to aggregate in cells [9], and that synthetic arginylated α-syn constructs have reduced aggregation properties in vitro [13]. Our current data expand on this finding, showing that arginylation specifically at E46 and E83, individually or together, reduces the formation of intraneuronal inclusions. Notably, this assay measures the seeding capacity of the α-syn fibrils added to the cells, which induce aggregation of intracellular α-syn. Thus, a reduction in the aggregate size following the seeding can in principle result not only from a reduction of the seeding capacity of arginylated α-syn, but also from a reduction in its cellular uptake. Investigating this additional possibility constitutes an exciting direction of further studies.

It is possible that arginylation also targets other α-syn sites, which may facilitate this effect in vivo and/or serve additional or different physiological roles. However, in vivo detection of arginylation by mass spectrometry still presents a challenge, even in the cases where other methods suggest the presence of highly arginylated protein fraction, making it difficult to definitively test this hypothesis. This phenomenon, and the potential existence and role of other arginylation sites in the brain, require further investigation.

PTMs are an emerging field, and very little is known about PTM hierarchy and their potential interactions with each other. Our work sheds light on such interactions by showing that the same protein pool is modified by multiple PTMs and that these PTMs can compete with each other in a hierarchical manner.

## Conclusions

In conclusion, the present study found that (1) α-syn is arginylated in the human brain on two conserved, functionally important sites – E46, previously implicated in familial PD, and E83, previously found to be critically important to α-syn pathology; (2) E46 and E83 arginylation targets a different percentage of α-syn in different patients, ranging from a few percent to nearly 50% of the total α-syn pool; (3) α-syn arginylation on these two sites strongly decreases with age, and negatively impacts α-syn pathological intracellular aggregation; and (4) α-syn arginylation counteracts S129 phosphorylation.

## Supplementary Information

**Additional file 1. Table S1**: Clinical profiles of donors with and without PD. **Table S2**: Primary antibodies used for western blot. **Fig. S1**: Characterization of E46- and E83-arginylated α-syn antibodies. **Fig. S2**: Both bands in the doublet recognized with α-syn antibodies contain α-syn. **Fig. S3**: S129 phosphorylation and E46 and E83 arginylation shows negative correlation with total α-syn levels. **Fig. S4**: E46 and E83 arginylation in the human brain decreases with patient age. **Fig. S5**: E46 and E83 arginylation levels show no correlation with ATE1 levels. **Fig. S6**: E46 and E83 arginylation alters the morphology and size of intracellular inclusions seeded by α-syn in cultured neurons. **Fig. S7**: E46 and E83 arginylation alters the morphology and size of intracellular inclusions seeded by a-syn in cultured neurons. **Fig. S8:** E46 and E83 5% arginylated a-syn fibrils are morphologically similar to wild type.

**Additional file 2. Dataset 1**: HPLC conditions and analysis for the synthetic peptides used for antibody generation

## Data Availability

All data and materials are included with the manuscript.

## Abbreviations

α-syn: α-Synuclein
PD: Parkinson’s disease
LB: Lewy bodies
PTM: Posttranslational modifications
ATE1: Arginyl transfer enzyme 1
