# Supplementary material for: α-Synuclein arginylation in the human brain
Source: Transl Neurodegener. 2022 Apr 8;11:20. doi: 10.1186/s40035-022-00295-0 (PMC8991655; doi:10.1186/s40035-022-00295-0)
Supplement: Supplementary file 1 — Additional file 1. Table S1: Clinical profiles of donors with and without PD. Table S2: Primary antibodies used for western blot. Fig. S1: Characterization of E46- and E83-arginylated α-syn antibodies. Fig. S2: Both bands in the doublet recognized with α-syn antibodies contain α-syn. Fig. S3: S129 phosphorylation and E46 and E83 arginylation shows negative correlation with total α-syn levels. Fig. S4: E46 and E83 arginylation in the human brain decreases with patient age. Fig. S5: E46 and E83 arginylation levels show no correlation with ATE1 levels. Fig. S6: E46 and E83 arginylation alters the morphology and size of intracellular inclusions seeded by α-syn in cultured neurons. Fig. S7: E46 and E83 arginylation alters the morphology and size of intracellular inclusions seeded by a-syn in cultured neurons. Fig. S8: E46 and E83 5% arginylated a-syn fibrils are morphologically similar to wild type. [file 40035_2022_295_MOESM1_ESM.docx]

**Supplemental Online Information**

**α−synuclein arginylation in the human brain**

Jun Zhao^1^, Buyan Pan^2^, Marie Fina^1^, Yun Huang^1^, Marie Shimogawa^2^, Kelvin C. Luk^3^, Elizabeth Rhoades^2^, E. James Petersson^2^, Dawei W. Dong^1^, and Anna Kashina^1*^

^1^Department of Biomedical Sciences, University of Pennsylvania School of Veterinary Medicine, ^2^Department of Chemistry, University of Pennsylvania School of Arts and Sciences, ^3^Center for Neurodegenerative Disease Research, Department of Pathology and Laboratory Medicine, University of Pennsylvania Perelman School of Medicine, Philadelphia, 19104, USA

*Corresponding author. E-mail: [akashina@upenn.edu](mailto:akashina@upenn.edu)

**Supplementary Table 1. Clinical profiles of donors with and without PD**

| **INDDID** | **Sex** | **Age** | **NPDx1** | **Label** |
| --- | --- | --- | --- | --- |
| 117849 | Male | 57 | Normal | C1 |
| 117504 | Male | 59 | Normal | C2 |
| 117843 | Male | 62 | Normal | C3 |
| 101060 | Male | 72 | Normal | C4 |
| 106373 | Male | 81 | Normal | C5 |
| 112568 | Male | 71 | PD | PD1 |
| 104020 | Male | 76 | PD | PD2 |
| 100604 | Male | 76 | PD | PD3 |
| 116261 | Male | 77 | PD | PD4 |
| 106324 | Male | 81 | PD | PD5 |
| 108180 | Male | 59 | PDD | PDD1 |
| 113669 | Male | 70 | PDD | PDD2 |
| 108649 | Male | 72 | PDD | PDD3 |
| 109022 | Male | 75 | PDD | PDD4 |
| 116441 | Male | 82 | PDD | PDD5 |

**Supplementary Table 2. Primary antibodies used for western blot**

| **Peptide/protein target** | **Host** | **Dilution used** | **Manufacturer** |
| --- | --- | --- | --- |
| GAPDH | Mouse  Monoclonal | 1:3000 | Proteintech Group Inc |
| a-synuclein | Rabbit  Polyclonal | 1:500 | Proteintech Group Inc (10842-1-AP) |
| S129 | Rabit  Polyclonal | 1:1000 | Abcam (ab51253) |
| E46 | Rabbit  Polyclonal | 1:200 |  |
| E83 | Rabiit  Polyclonal | 1:200 |  |
| ATE1 | Rat  Polyclonal | 1:1000 |  |

**Zhao_et_al_FigS1**


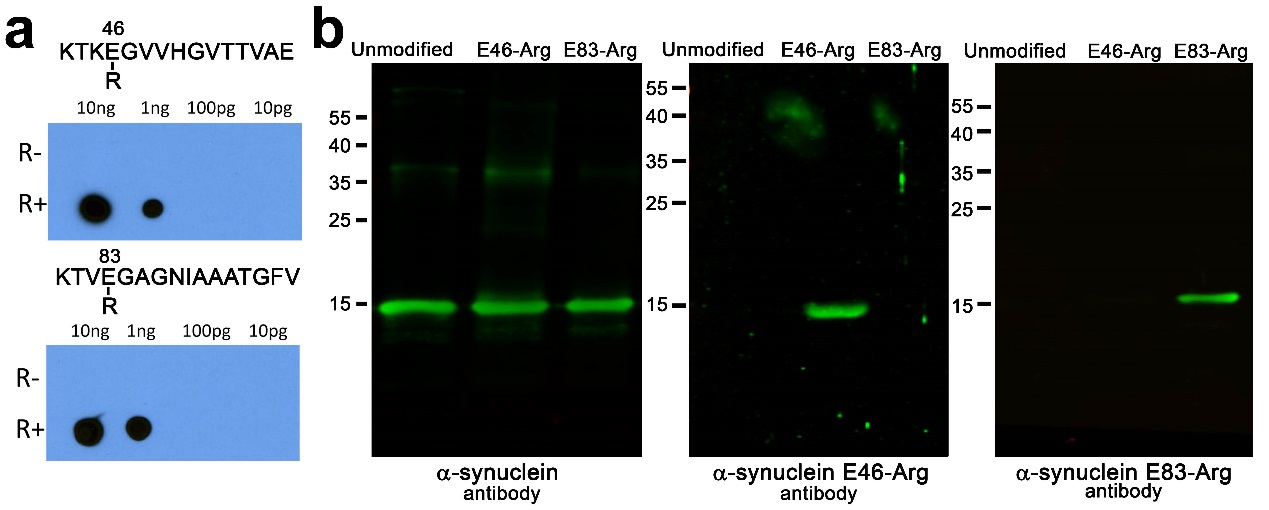


**Fig. S1. Characterization of E46- and E83-arginylated α−syn antibodies.** a, dot blots with the E46 (top) and E83 (bottom) antibodies against the synthetic peptides that were used for antibody production (shown on top, denoted with R+ on the side of the blot) and the non-arginylated control peptides (same sequence without the R on the side chain, denoted with R- on the side of the blot). Peptide concentrations loaded on the blot are shown on top. b, representative Western blots of standard unmodified or arginylated α−syn, showing antibody specificity to the appropriate α−syn variants.

**Zhao_et_al_FigS2**


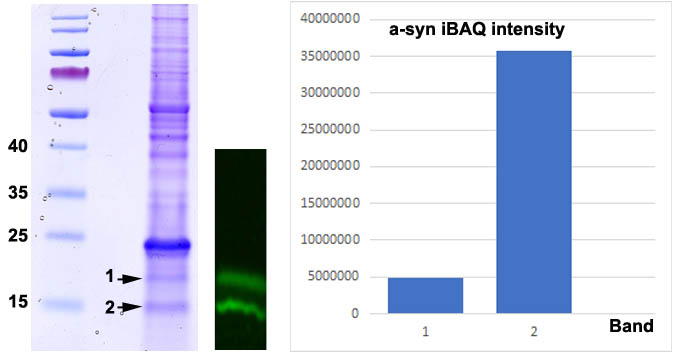


**Fig. S2. Both bands in the doublet recognized with α−syn antibodies contain α−syn.** SDS gel used for band excision (left) is shown next to a scaled Western blot of the band doublet (middle) and the iBAQ intensity of the a-syn containing peptides in each band.

**Zhao_et_al_FigS3**


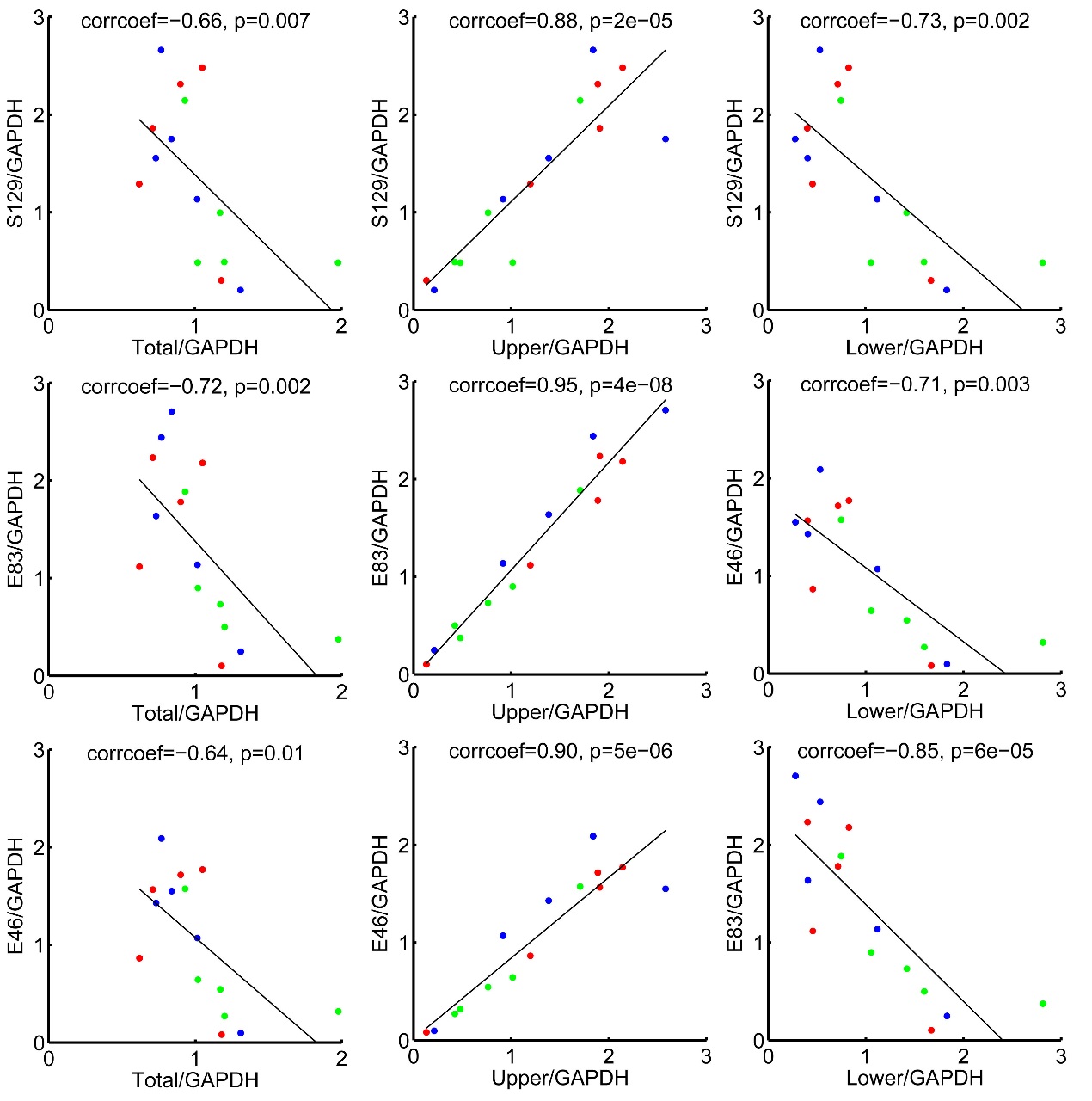


**Fig. S3. S129 phosphorylation and E46 and E83 arginylation shows negative correlation with total α−syn levels.** Correlation plots between different antibody signals calculated from the same datasets as those shown in Fig. 1. Spearman correlation coefficient and p-value are listed on top of each plot. The p-values were calculated by transforming the correlation between n pairs of concerned variables to create a t-statistic having n-2 degrees of freedom.

**Zhao_et_al_FigS4**


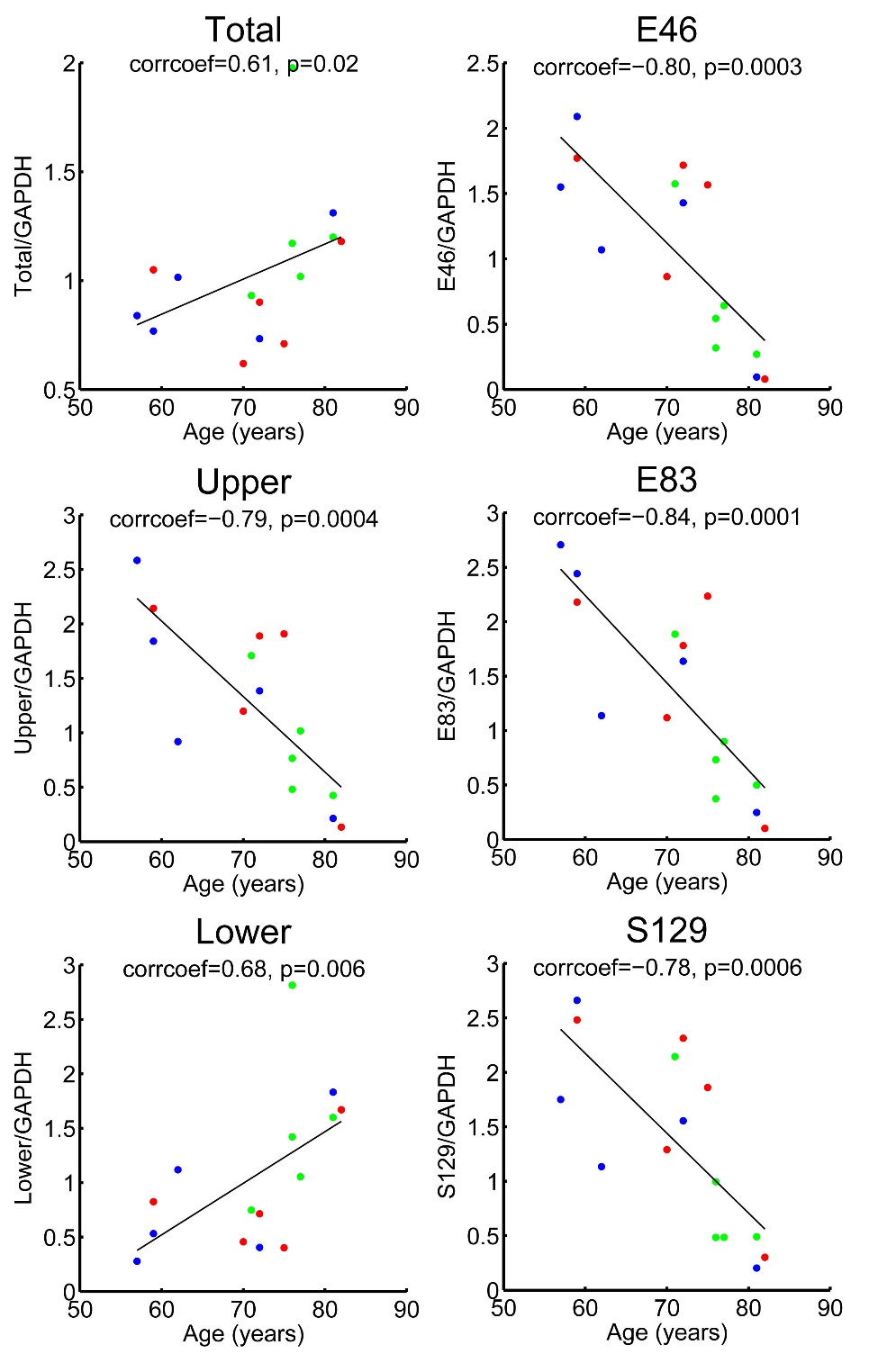


**Fig. S4. E46 and E83 arginylation in the human brain decreases with patient age.** Correlation plots between different antibody signals calculated from the same datasets as those shown in Fig. 1. Spearman correlation coefficient and p-value are listed on top of each plot. The p-values were calculated by transforming the correlation between n pairs of concerned variables to create a t-statistic having n-2 degrees of freedom.

**Zhao_et_al_FigS5**


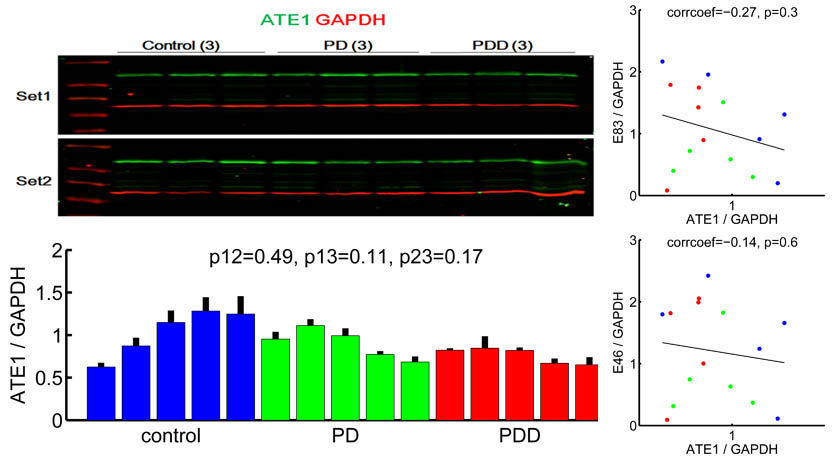


**Fig S5. E46 and E83 arginylation levels show no correlation with ATE1 levels.** Representative Western blots, quantification (SEM, n=3), and correlation plots are shown. Pearson correlation coefficient and p-value are listed on top of each plot. The p-values were calculated by transforming the correlation between n pairs of concerned variables to create a t-statistic having n-2 degrees of freedom.

**Zhao_et_al_FigS6**


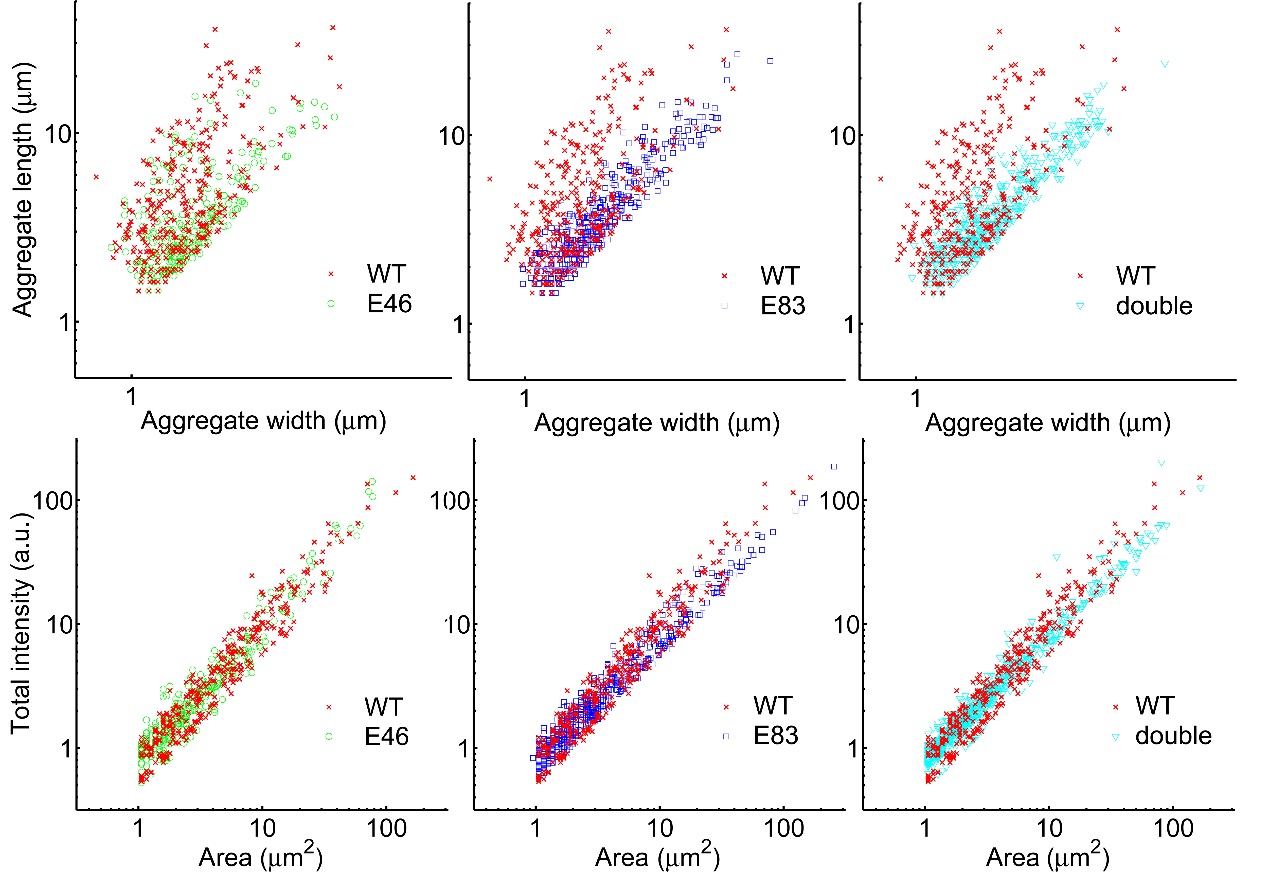


**Fig S6. E46 and E83 arginylation alters the morphology and size of intracellular inclusions seeded by α−syn in cultured neurons.** Scatter plots of the data shown in Fig. 6 of the main text, plotted as individual data points correlated to relative measurements of the same aggregates. N=50 independent fields of view.

**Zhao_et_al_FigS7**


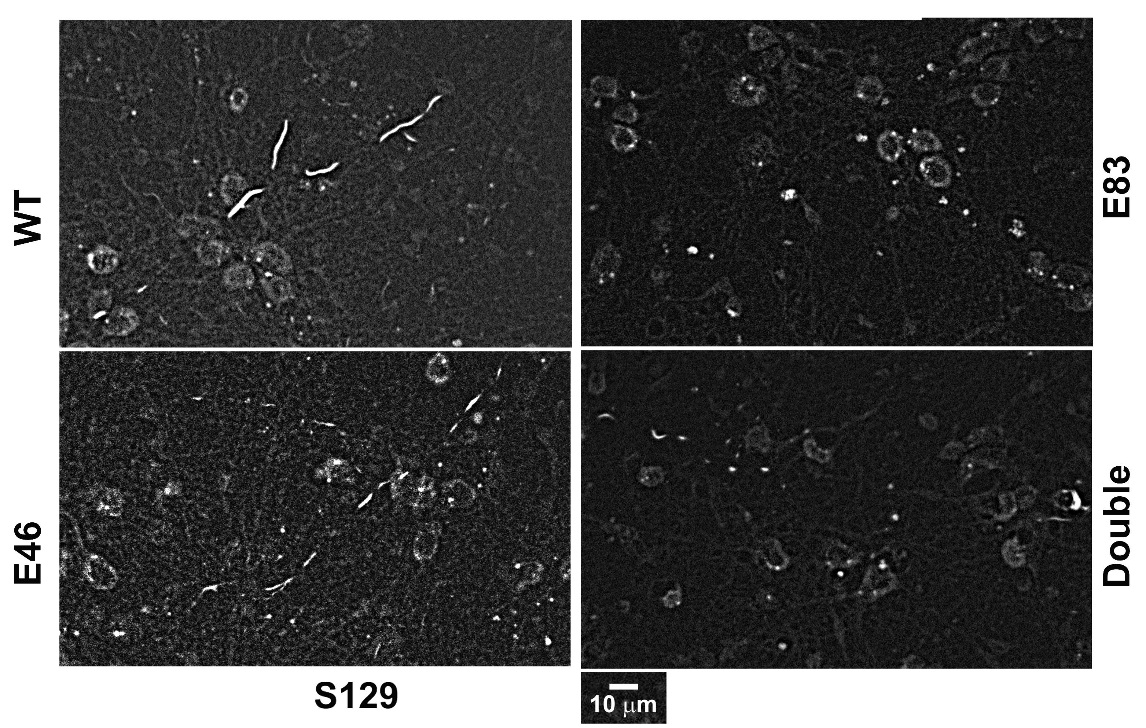


**Fig S7. E46 and E83 arginylation alters the morphology and size of intracellular inclusions seeded by a-syn in cultured neurons.** Representative images of the intracellular aggregates stained with S129 antibodies are shown. Scale bar, 10 μm.

**Zhao_et_al_FigS8**


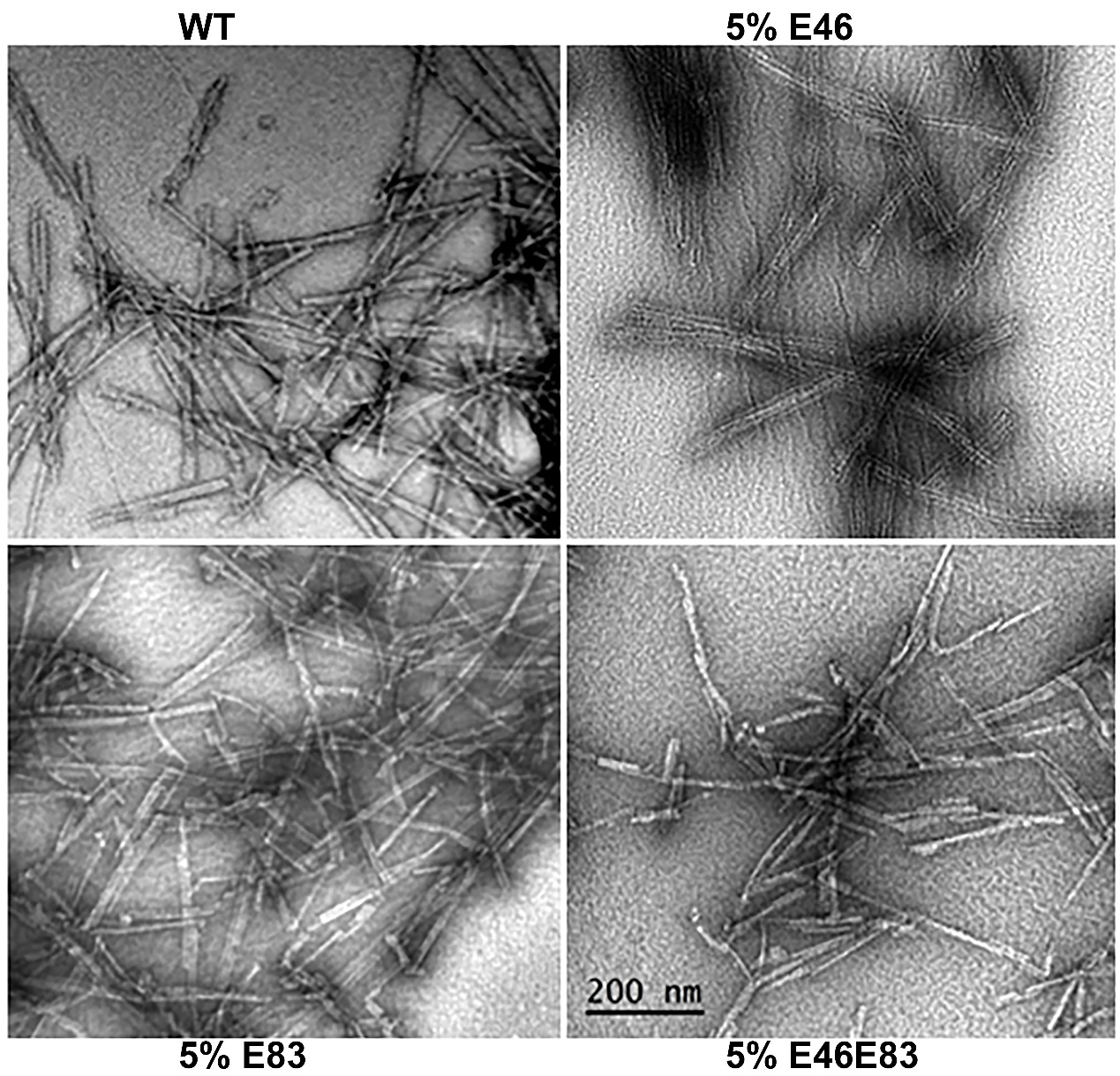


**Fig S8. E46 and E83 5% arginylated a-syn fibrils are morphologically similar to wild type.** Representative negative staining electron microscopy images of the a-syn fibrils, either non-arginylated (WT) or arginylated as labeled on the panels. Scale bar, 200 nm.
