# Supplementary material for: α-Synuclein arginylation in the human brain
Source: Transl Neurodegener. 2022 Apr 8;11:20. doi: 10.1186/s40035-022-00295-0 (PMC8991655; doi:10.1186/s40035-022-00295-0)
Supplement: Supplementary file 2 — Additional file 2. Dataset 1: HPLC conditions and analysis for the synthetic peptides used for antibody generation [file 40035_2022_295_MOESM2_ESM.pdf]

All HPLC condition is:

Column: Phenomenex Luna C8 5 μm, 150 mm × 4.6 mm

Solvent: A: 0.1% TFA/H_2O; B: 0.1% TFA/Acetonitrile

Gradient: 2%-60%B, 30 min, 1ml/min

CVGSKTKE(R)GVVH

MALDI : Calcd. 1399.75, found. 1399.69 [MH]+, 1421.67 [M+Na]+

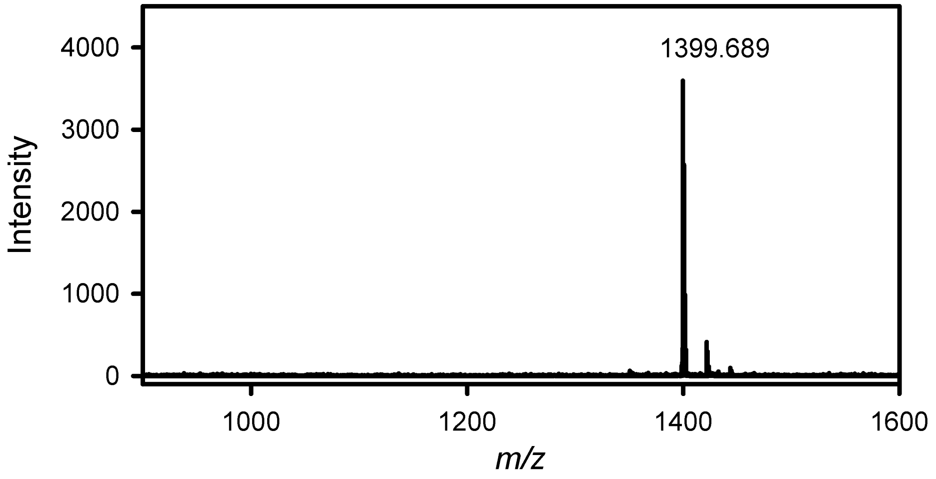

HPLC

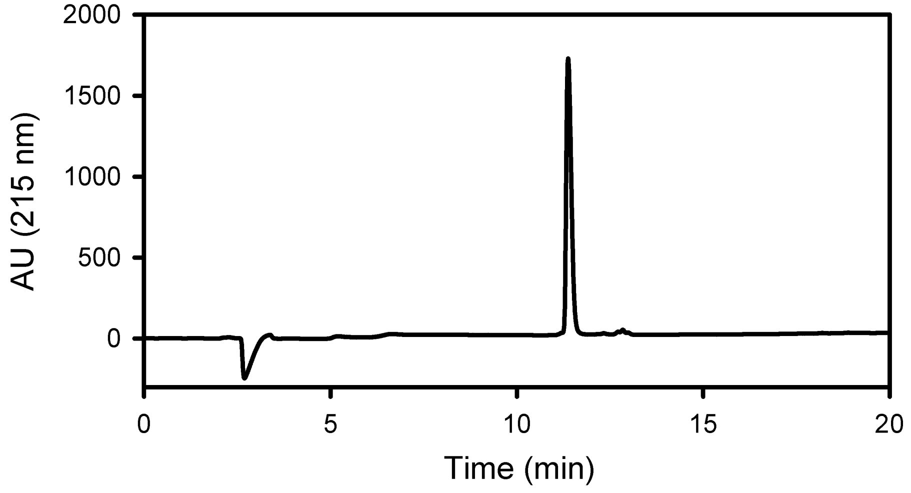

t_R = 11.37 min

CAVAQKTVE(R)GAG   (G + Arg peptide)

MALDI: Calcd. 1189.66, found. 1289.93 [MH]+, 1311.91 [M+Na]+

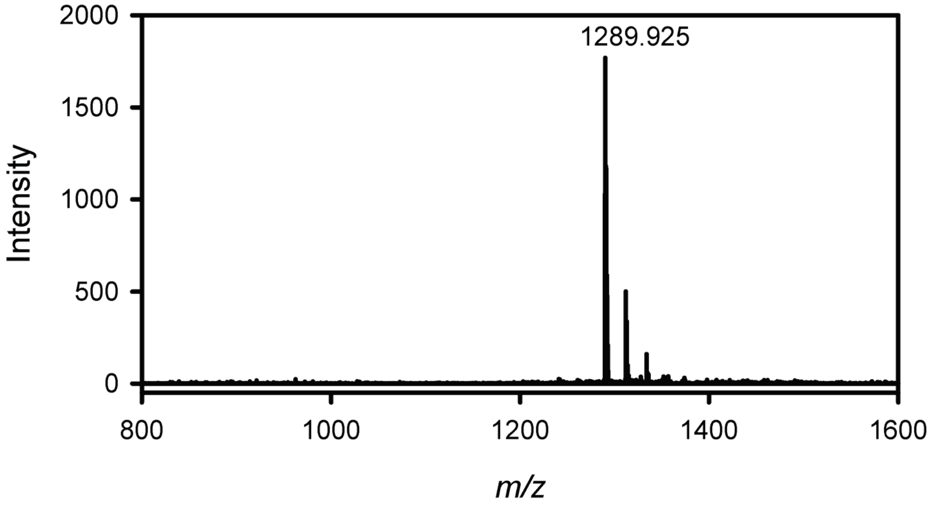

HPLC

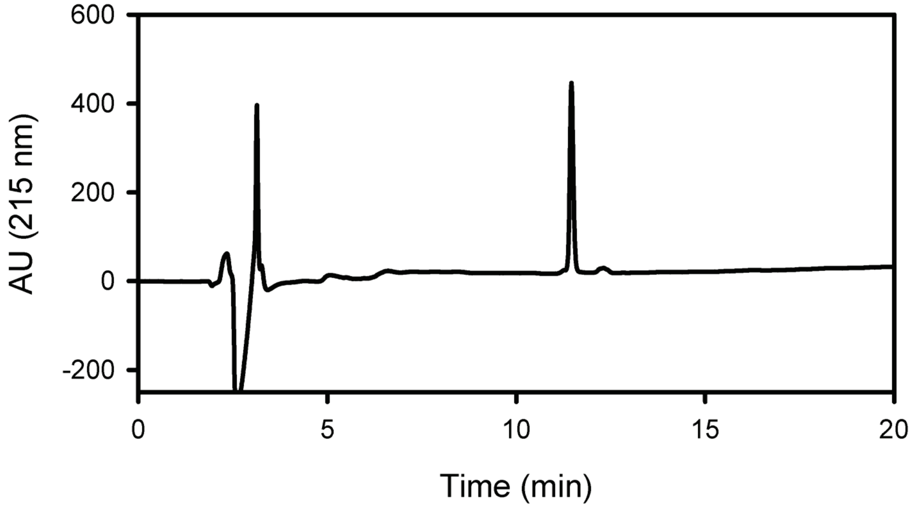

t_R = 11.46 min
